# Evoked Temporal Summation in Cats to Highlight Central Sensitization Related to Osteoarthritis-Associated Chronic Pain: A Preliminary Study

**DOI:** 10.1371/journal.pone.0097347

**Published:** 2014-05-23

**Authors:** Martin Guillot, Polly M. Taylor, Pascale Rialland, Mary P. Klinck, Johanne Martel-Pelletier, Jean-Pierre Pelletier, Eric Troncy

**Affiliations:** 1 Groupe de Recherche en Pharmacologie Animale du Québec (GREPAQ), Department of Biomedical Sciences, Faculty of Veterinary Medicine – Université de Montréal, Saint-Hyacinthe, Quebec, Canada; 2 Osteoarthritis Research Unit, Université de Montréal Hospital Centre, Notre-Dame Hospital, Montreal, Quebec, Canada; 3 Topcat Metrology, Gravel Head Farm, Downham Common, Little Downham, Nr Ely, Cambridgeshire, United Kingdom; The James Cook University Hospital, United Kingdom

## Abstract

In cats, osteoarthritis causes significant chronic pain. Chronicity of pain is associated with changes in the central nervous system related to central sensitization, which have to be quantified. Our objectives were 1) to develop a quantitative sensory testing device in cats for applying repetitive mechanical stimuli that would evoke temporal summation; 2) to determine the sensitivity of this test to osteoarthritis-associated pain, and 3) to examine the possible correlation between the quantitative sensory testing and assessment using other pain evaluation methods. We hypothesized that mechanical sub-threshold repetitive stimuli would evoke temporal summation, and that cats with osteoarthritis would show a faster response. A blinded longitudinal study was performed in 4 non-osteoarthritis cats and 10 cats with naturally occurring osteoarthritis. Quantification of chronic osteoarthritis pain-related disability was performed over a two week period using peak vertical force kinetic measurement, motor activity intensity assessment and von Frey anesthesiometer-induced paw withdrawal threshold testing. The cats afflicted with osteoarthritis demonstrated characteristic findings consistent with osteoarthritis-associated chronic pain. After a 14-day acclimation period, repetitive mechanical sub-threshold stimuli were applied using a purpose-developed device. Four stimulation profiles of predetermined intensity, duration and time interval were applied randomly four times during a four-day period. The stimulation profiles were different (*P*<0.001): the higher the intensity of the stimulus, the sooner it produced a consistent painful response. The cats afflicted with osteoarthritis responded more rapidly than cats osteoarthritis free (*P* = 0.019). There was a positive correlation between the von Frey anesthesiometer-induced paw withdrawal threshold and the response to stimulation profiles #2 (2N/0.4 Hz) and #4 (2N/0.4 Hz): Rho_s_ = 0.64 (*P* = 0.01) and 0.63 (*P* = 0.02) respectively. This study is the first report of mechanical temporal summation in awake cats. Our results suggest that central sensitization develops in cats with naturally occurring osteoarthritis, providing an opportunity to improve translational research in osteoarthritis-associated chronic pain.

## Introduction

Feline osteoarthritis (OA) develops with ageing in diarthrodial joints, predominantly the elbow, coxofemoral and stifle joints, and causes chronic pain [1]–[4]. Assessment of chronic pain in animals with OA takes into account the impact of pain on both physical ability and quality of life. Owners of cats with OA observe a number of altered behaviour patterns such as decreased daily activity and a reluctance to jump or to walk up stairs [5]–[8]. Objective functional methods have also been developed to evaluate OA-associated disability in cats. The peak vertical ground reaction force (PVF) quantifies limb impairment that may be related to decreased use because of pain [9]–[11]. In addition, accelerometer-based motor activity (MA) assessment enables objective quantification of the impairment of normal function related to OA-associated chronic pain [10], [12], [13].

Central sensitization is expressed as pain hypersensitivity, particularly dynamic tactile allodynia, secondary punctate hyperalgesia, aftersensations, and enhanced temporal summation (TS) [14] and is present in OA [15], [16]. Quantitative sensory testing (QST) is used to characterize these abnormal sensations [17]. Until recently, little attention had been paid to detailed assessment of sensory abnormalities in animals. Neurology texts describe the gross assessment of sensory function (*e.g.*, the response to pinching skin in various dermatomes) [18] and pain management texts refer to the theory of altered sensory processing (peripheral and central sensitization) associated with acute injury or chronic disease [18]. Most information on QST is based on rodent models and the human literature; in particular regarding the (mechanical) pressure pain threshold in painful OA [19], [20]. Several studies in humans suggest that QST to detect mechanical allodynia and hyperalgesia should be an integral part of the assessment of OA-associated chronic pain [15], [19]–[23]. Quantitative ST using von Frey anesthesiometer-induced paw withdrawal threshold (vFWT) in dogs [22] and cats [10] with natural OA represents one of the first attempts to evaluate the changes in central processing in companion animals suffering chronic pain.

Low frequency repetition of a fixed-intensity stimulus increases the action potential discharge of dorsal horn neurons followed by after discharges; this activity-dependent facilitation is called spinal windup [24]. The early phase of windup is denoted as TS, and has been widely used to investigate spinal cord excitability [14], [25]. Windup is considered to be an intrinsic part of the early plastic changes in the central nervous system leading to chronicity, hence TS is an ideal target for studying chronic pain [14].

Our objectives were 1) to develop a QST device in cats for applying repetitive mechanical stimuli that would evoke TS; 2) to determine the sensitivity to OA pain (discriminatory ability) of a number of stimulation profiles, and 3) to examine the correlation between the repetitive stimuli QST responses and assessment using other objective chronic pain evaluation methods (PVF, MA monitoring, and vFWT).

We hypothesized that mechanical sub-threshold repetitive stimuli QST with a controlled profile would evoke TS, and that cats with OA would show an enhanced response.

This study aimed to provide insight about central sensitization in cats with naturally occurring OA. Using an innovative QST technique, this study results suggest that central sensitization is a feature of feline OA-associated chronic pain.

## Methods

### Ethics statement

The Institutional Animal Care and Use Committee (*Comité d'Éthique et d'utilisation des Animaux* (CÉUA) of *Université de Montréal*) approved the study protocol (# Rech-1482), and the Canadian Council on Animal Care guidelines were followed for all cat care and procedures undertaken. Furthermore, this study adhered to the guidelines of the Committee for Research Ethical Issues of the IASP [26], and the ARRIVE guidelines for reporting animal research [27].

### Animals and experimental design

This study used 4 normal healthy, non-OA cats (one neutered female, and three neutered males), and 10 cats with naturally occurring OA (5 neutered females, and 5 neutered males) belonging to the colony of a contract research organization (ArthroLab, Inc., Montreal, QC, Canada) accredited by the Canadian Council on Animal Care. The cats were housed together in a dedicated room (floor area approximately 8×12 m). The room's environment and the cats' health were monitored and recorded daily. The cats were fed a standard certified commercial cat food (Hill's Prescription Diet w/d Feline, Hill's Pet Nutrition, Inc., Mississauga, ON, Canada) once daily in the afternoon according to the food manufacturer's recommendations. Water was supplied *ad libitum*. The cats were loose-housed, with free access to toys, raised platforms, and a large window. Beds in a quiet area were also freely accessible.

No abnormalities were detected upon neurologic evaluation, complete blood count, blood biochemical profile (including T4), and urine analysis; nor were there any limb deformities or signs of acute musculoskeletal disease. All cats were free from both feline immunodeficiency and leukemia viruses. The extent of radiographic OA was graded by a veterinary radiologist as previously described [9]–[11], , using computed radiographs of the stifle, coxofemoral, carpal and tarsal joints (mediolateral and caudocranial projections), and of the shoulders and elbows joints (mediolateral projections). These radiographs were performed under sedation using medetomidine (0.02 mg/kg; Domitor 1 mg/mL, Zoetis Canada, Kirkland, QC, Canada) and morphine (0.1–0.2 mg/kg; Morphine Sulfate Injection 10 mg/mL, Sandoz, Boucherville, QC, Canada), administered intramuscularly.

On the first day of this longitudinal study, a certified veterinary behaviourist, blinded to the radiographic grade and age of the cats, performed a behavioural examination. This examination aimed to detect changes in gait, posture, and the presence of subjective joint pain. Cats designated “non-OA” had no abnormalities detected during this examination, and selected “OA cats” were considered subjectively to be in pain (refer to Table 1).

**Table 1:** Age, body weight, radiographic and clinical features of the selected cats.

Features		Non-OA cats	OA cats
Mean age (range; year)		**3.4 (1.5–4.5)**	**9.3 (7.0–12.0)**
Mean body weight (range; kg)		5.1 (3.5–7.1)	4.8 (3.1–6.2)
Median radiographic scores (range)	Forelimbs	0 (0–0)	2 (0–4)
	Hind limbs	0 (0–0)	2 (0–9)
Median radiographic OA-affected joint number (range)	Forelimbs	0 (0–0)	1 (0–2) a
	Hind limbs	0 (0–0)	2 (0–4) b
Presence of gait alteration		**0/4**	**5/10**
Presence of posture alteration	Forelimbs	0/4	1/10
	Hind limbs	**0/4**	**4/10**
Presence of subjective pain	Forelimbs	0/4	2/10
	Hind limbs	**0/4**	**10/10**

a Affected joints were shoulder (6/10), elbow (5/10), and carpal (1/10) joints.

b Affected joints were coxofemoral (9/10), stifle (5/10), and tarsal (5/10) joints.

The cats were acclimated and trained for one week only immediately prior to the study, as they already had six months experience with all the procedures except repetitive stimuli QST. Chronic OA pain-related disability was quantified over a two-week period using functional methods that consisted of PVF measurement and MA intensity assessment. During the same period, objective chronic pain evaluation was undertaken using mechanical QST with vFWT testing to assess secondary punctate tactile allodynia [10]. Finally, several repeated mechanical stimuli QST protocols were tested after a further 14-day acclimation period. This comprised positive reinforcement for progressive habituation to the evaluation environment: being in the evaluation cage, wearing the stimulation device, and being stimulated by the device.

### Measurement of PVF

The cats' PVF were recorded twice, a week apart. Measurements were performed within 60 sec of approximately 3 minutes of stair exercise. This consisted of running up and down, and again up a 10 m long staircase; this acquisition protocol decreases data variability and optimizes effect sizes [11]. Post-exercise PVF were acquired using a floor mat-based plantar force measurement system (Walkway System WE4, Tekscan Inc., Boston, MA) while the cats trotted across the walkway at a comfortable speed (0.8–1.4 m/sec). Speed was computed by the software, using length and duration of a given stride. Equilibration and calibration of the system were performed prior to each acquisition session, as previously described [9]–[11]. A maximum of 3 valid trials (with the cat moving across the entire mattress undisturbed, consistently, in a straight line, and at the correct speed) were obtained for each cat, with an *a priori* maximum of 16 consecutive trials allowed. The number of trials needed to obtain the 3 valid trials was recorded [10].

The analysis focused on the hind limbs, as the subjective pain evaluation indicated that these were the only painful limbs. The PVF data management used the most affected hind limb, determined as the hind limb that generated the lower PVF value [expressed in % body weight (BW)] most frequently (maximum of 2×3 = 6 trials) [11]. If an equal number of lower values was detected for each hind limb, the hind limb with the lower average PVF was chosen.

### Motor activity assessment

The MA intensity was assessed using a collar-mounted accelerometer-based activity sensor (ActiWatch, Minimitter/Respironics, distributed by Bio-Lynx Scientific Equipment Inc., Montreal, QC, Canada) maintained in place for two weeks. The device was set for local time and configured to create 1 count per 2 minutes. The amplitude of each count was subsequently translated into a numeric value (from 0 to infinite) describing the MA intensity. In common with previous studies [9], [10], and to avoid the effects of human interference, analysis of the cats' activity was restricted to 3 days per week (Friday, Saturday and Sunday), between 5:00 pm and 7:00 am. Data were expressed as the average total intensity count. The final MA intensity was calculated for each cat by taking the median of the three days recorded.

### Secondary punctate tactile allodynia quantification

**Figure 1:**
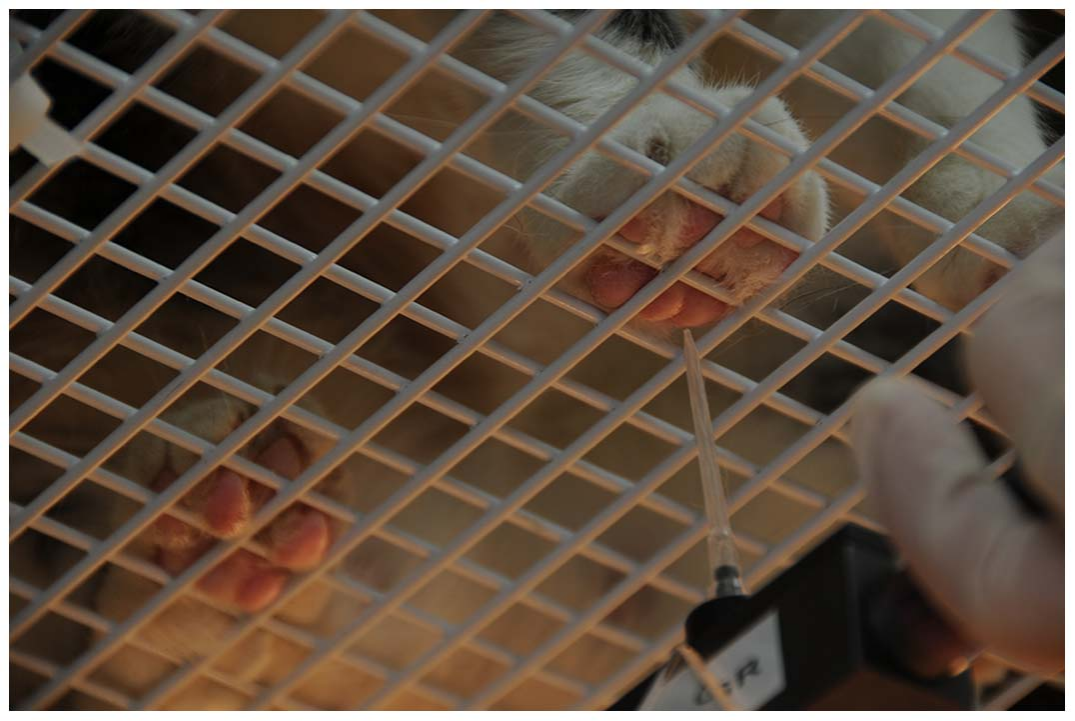
Photograph of the placement of the von Frey probe. During evaluation, the cats stood in a meshed cage, the probe tip was placed perpendicular to the plantar surface of the foot, and an increasing force was applied without manipulating the limb.

Secondary punctate allodynia was quantified twice, at a week's interval, using a mechanical von Frey polypropylene probe (Rigid Tip, surface area 0.7 mm^2^, IITC Life Science, Woodland Hills, CA) fitted onto the hand-held force transducer of a paw withdrawal threshold monitoring anesthesiometer. With the cat standing in a meshed cage (Model 55035, Hunter Brand, Inc., Montreal, QC, Canada; dimensions 33” ×22” ×37”), the probe tip was placed perpendicular to the plantar surface of the foot (Fig. 1), and an increasing force was applied without manipulating the limb. The four limbs were tested in a predefined order with a 60-s interval between stimuli. The same evaluator performed all evaluations, and was blinded to the cat's OA status. The stimulus was stopped as soon as the paw was withdrawn, and the peak force recorded. Duplicate measurements were obtained from each paw. Data under 2 g were discarded, and a maximum cut off value of 200 g was applied. For both evaluations (one week apart), the vFWT was expressed as the average of the available threshold values (maximum of 2×4 = 8 values).

### Mechanical repetitive stimuli quantitative sensory testing

**Figure 2:**
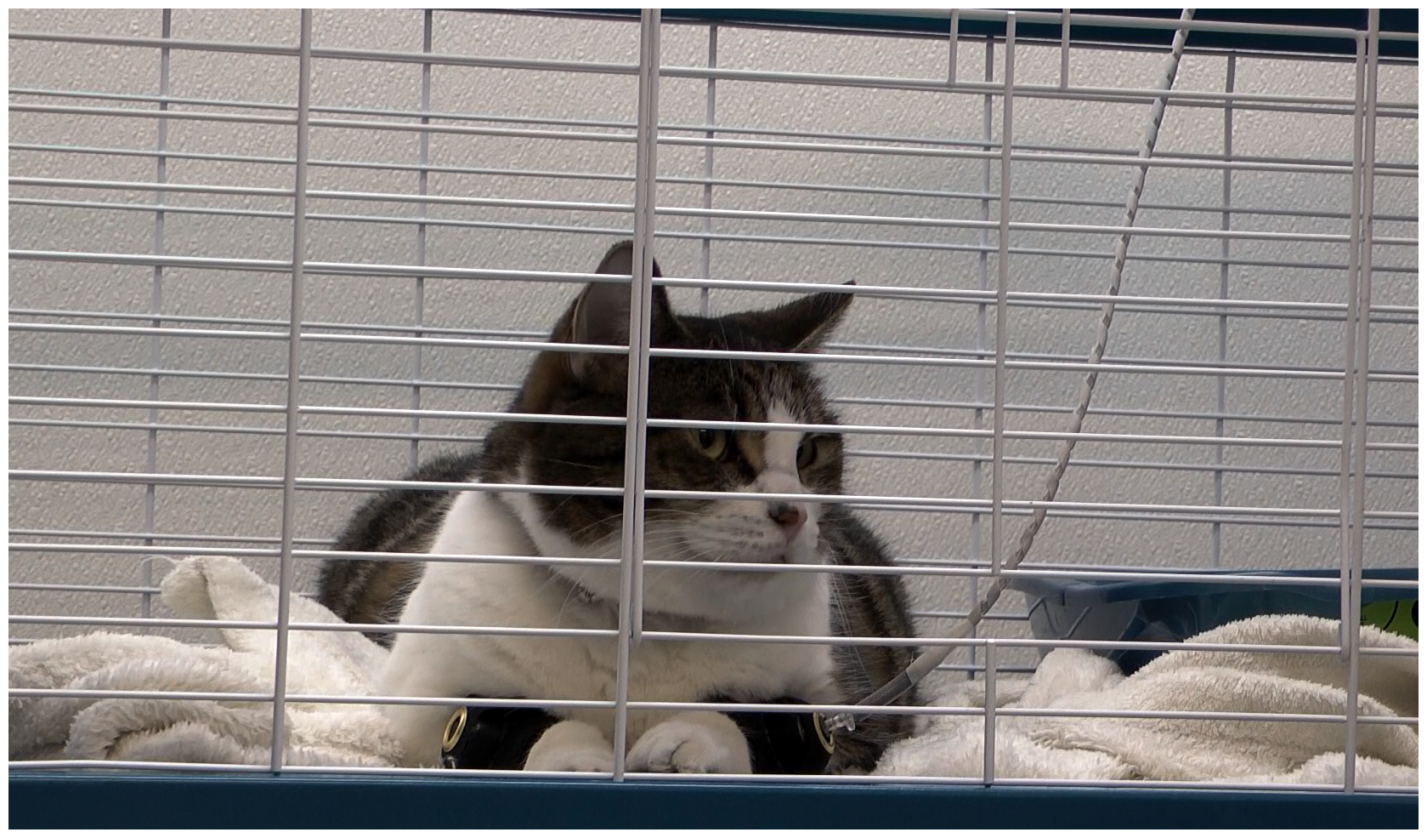
Photograph of the mechanical repetitive stimuli quantitative sensory testing experimental setting. Cats were placed in a meshed cage. The mechanical stimulator, which was embedded in a small band, was placed around the distal aspect of the cat's foreleg (left in this photograph) and connected to the stimulator device, while a dummy band was installed on the contralateral leg (right in this photograph).

Repeated mechanical stimuli of sub-threshold intensity (that is, for which a single stimulus would not elicit pain behaviour) were applied using a purpose-made device (Topcat Metrology Ltd; Cambs, UK). Available protocols of TS in humans and animals were used to devise the stimulation set profiles used in this study [29]–[32], and the profiles were refined during a pilot study in three cats (2 with OA; data not shown). The device supplied repeated mechanical stimuli at a predetermined intensity, duration and time interval. The mechanical stimulus was produced by hemispherical-ended metallic pin (2.5 mm diameter, 10 mm length) mounted on a rolling diaphragm actuator, adapted from a validated mechanical threshold testing system [33]. The actuator was mounted on the anterolateral aspect of the right or left mid metacarpus, held by a narrow band around the leg; a dummy was installed on the contralateral leg. During testing, the cats were free to move about in the same meshed cage (Fig. 2).

Each cat underwent four separate testing sessions (two sessions on the right leg, and two on the left), each separated by one day. Two of these sessions were conducted in the morning, and two during the afternoon. Before each testing session, the evaluator spent 5 minutes watching the normal behaviour of the cat once it had been placed in the cage, wearing the stimulator device. During each testing session one series of each of four sets of stimulation profiles was completed in a randomized order, with a 5-minute interval between each set of stimuli. Each stimulation set comprised up to 30 stimuli with one the four profiles of intensity and/or frequency; the power of the stimulation set increased from profile #1 to 4 (Table 2). The evaluator was blinded to the cat's OA status and the stimulation profile. During each set of stimuli, testing was either stopped by the evaluator as soon as clear pain behaviour was seen (*e.g.* vocalization, agitation, biting at the limb band, vigorously shaking the leg or jumping away from it) or stimulation was stopped automatically when the maximum number of stimuli (30) was reached. The number of stimuli (NS) reached was noted for each test. The response to each stimulation profile was defined as the NS for each cat by taking the median of the four NS recorded for each stimulation profile.

**Table 2:** Characterization of the mechanical stimuli provided by each stimulation profile.

Stimulation profile	Intensity (N)	Duration (s)	Frequency (Hz)	Interval (s)	Maximal number
#1	2	1.5	0.4	2.5	30
#2	4	1.5	0.4	2.5	30
#3	6	1.5	0.125	8	30
#4	6	1.5	0.4	2.5	30

### Statistical methods

All analyses were two-sided with an α threshold of 0.05 using a statistical software program (SAS system, version 9.3, SAS Institute Inc., Cary, NC, USA). Continuous data distribution was assessed using the Shapiro-Wilk test (normal distribution) and kernel density estimation. The NS data sets comprised count data that were assumed to be Poisson distributed by nature.

Mixed model analyses for repeated measures were conducted to compare the PVF and vFWT between non-OA and OA cats [34], [35]. Generalized linear mixed model analyses for repeated measures were conducted using conditional models to compare the MA intensity (Weibull distributed data) between non-OA and OA cats, and to compare the responses of the mechanical repeated NS (Poisson distributed data) between stimulation profiles and between non-OA and OA cats [36], [37]. Whole model details are shown in Table 3. These models provided fixed effect estimates by restricted likelihood modelling. Homogeneity of variance was assessed using the absolute values of the residuals of the mixed model, and the best structure of the covariance model was assessed using a graphical method (plots of covariance *versus* lag in time between pairs of observations compared to different covariance models in mixed models), as well as using information criteria that measure the relative fit of competing covariance models (mixed models, and generalized linear mixed models). Also, residuals of the models were thoroughly studied to assess the model's validity. A Bonferroni adjustment provided adjusted p-values (adj-*P*), and adjusted 95% confidence interval (95% CI) for multiple comparisons when appropriate.

**Table 3:** Details of the mixed model analyses.

Data	Data distribution	Data transformation*	Fixed effects	Random effects	Covariance structures	Tested covariates
PVF	Normal	Log-transformed	Cat group, evaluation day, and cat group × evaluation day	Cat	Compound symmetry	Age, BW, Velocity and Maximum number of trials
MA	Weibulll	None		Cat, and evaluation day	Compound symmetry	Age, and BW
vFWT	Normal	Log-transformed		Cat	Type 1 autoregressive	
NS	Poisson	None	Cat group, stimulation profile, cat group × stimulation profile	Stimulation profile, and cat × stimulation profile	Compound symmetry	

^*^Outcome transformations were recommended following residual analysis results to correct for data heteroscedasticity; PVF: ; MA: motor activity; vFWT: von Frey anesthesiometer-induced paw withdrawal threshold; NS: number of stimuli, BW: body weight.

Exploratory correlations between PVF expressed as %BW, MA intensity, vFWT (the mean value of the two evaluations were used in this analysis for the three above outcomes), and the NS from the four stimulation profiles were carried out using Spearman's rank correlations.

## Results

**Figure 3:**
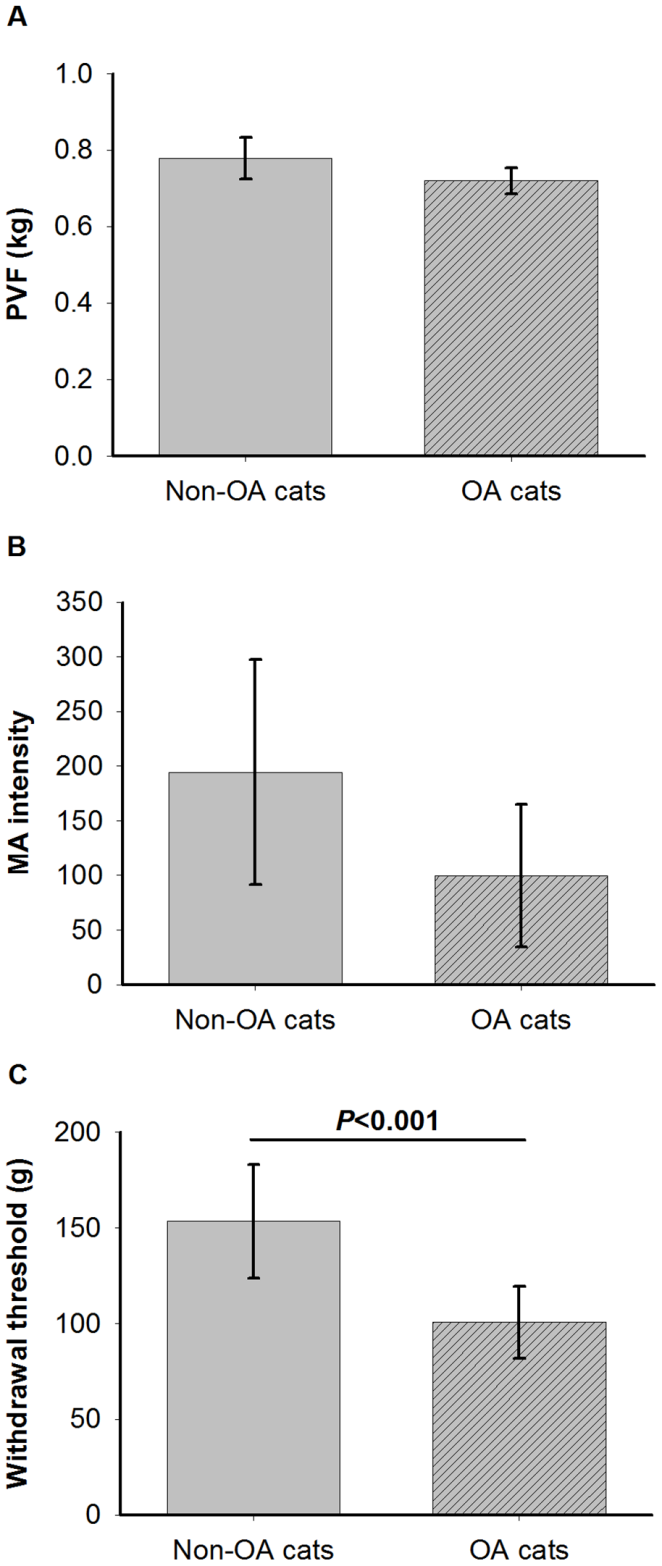
Characterization of osteoarthritis (OA) using chronic pain evaluation methods: A- Least squares means and 95% confidence interval of the log-transformed most affected limb peak vertical ground reaction force (PVF) after-exercise by OA status. B- Least squares means and 95% confidence interval of the motor activity (MA) intensity by OA status. C- Least squares means and 95% confidence interval of the log-transformed von Frey anesthesiometer-induced paw withdrawal threshold by OA status.

PVF after exercise in the most affected hindlimb tended to be lower in the OA cats compared with the non-OA cats (Fig. 3A; cat group effect *P* = 0.070): least squares means estimate difference (LSD; 95% CI)  =  −0.059 kg (−0.124, 0.005). PVF analyses also identified some significant covariates: BW (*P*<0.0001) and the inverse of the maximum number of trials (*P*<0.001). However, the evaluation day effect was not significant (*P* = 0.79), nor was there a significant interaction between cat group and evaluation day (*P = *0.41).

MA intensity was not significantly lower in OA cats than in non-OA cats (Fig. 3B; cat group effect *P* = 0.12): LSD (95% CI)  =  −95 (−216, 27). The analyses neither showed a significant evaluation day effect (*P* = 0.72) nor an interaction of cat group with evaluation day (*P* = 0.78).

vFWT was significantly lower in OA cats than in non-OA cats (Fig. 3C; cat group effect *P* = 0.007): LSD (95% CI)  =  −53 g (−88, −18). There was no significant evaluation day effect (*P* = 0.76) nor any interaction between cat group and evaluation day (*P* = 0.79).

**Figure 4:**
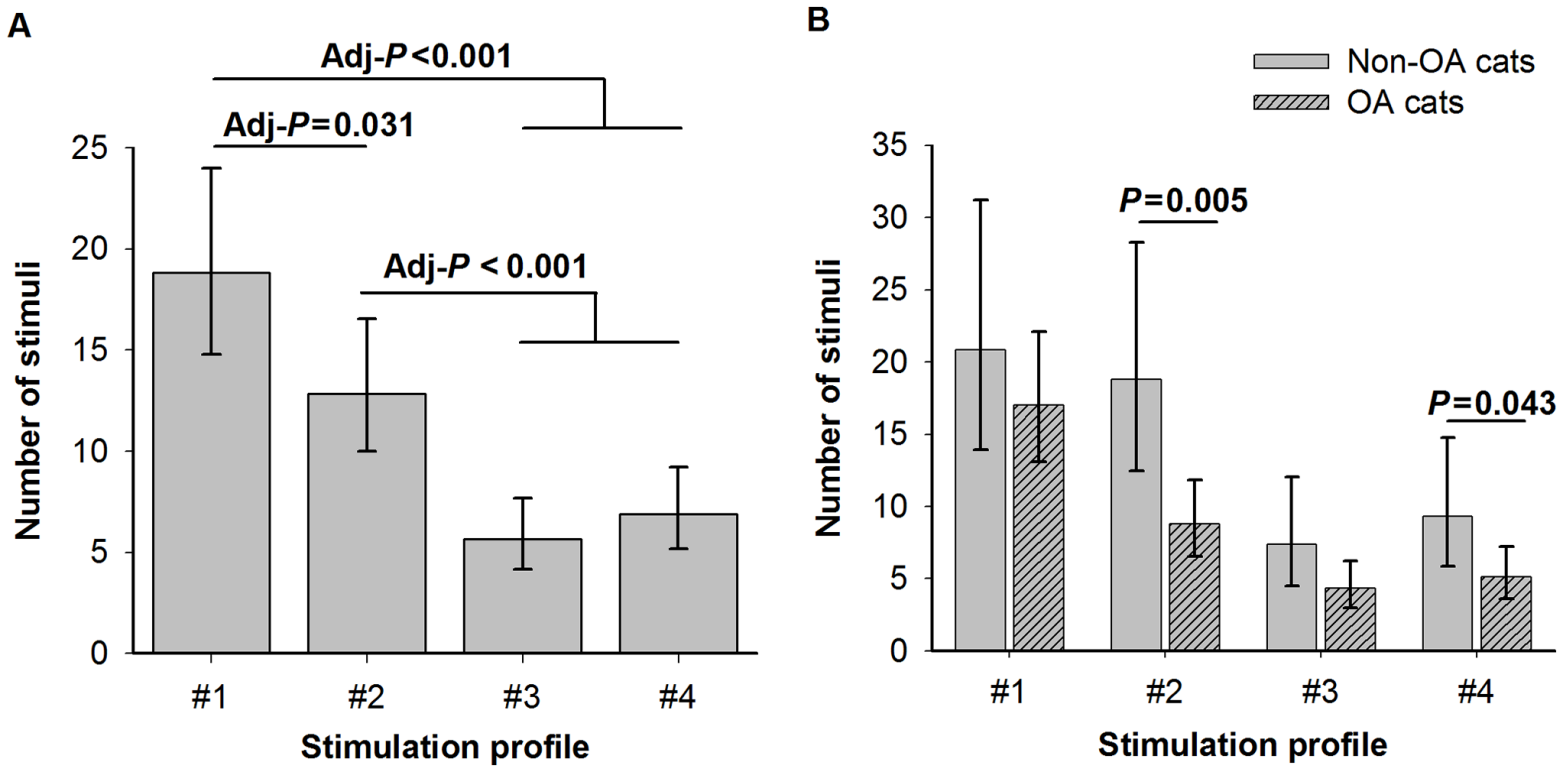
Number of stimuli reached and 95% confidence interval (inverse link of the least squares means estimates and 95% confidence interval obtained using the Poisson generalized linear modelling) following repetitive mechanical stimuli: A- by stimulation profiles (#1 to 4); B- by stimulation profiles and osteoarthritis (OA) status. Adj-*P* =  adjusted p-value.

Sustained pain behaviours persisting for several seconds after the end of the stimulation set were observed with all stimulation profiles. Analysis of the mechanical repetitive stimuli QST data identified significant differences between the profiles (stimulation set effect *P*<0.001): the higher the intensity of the stimulus, the sooner (lower NS) it produced a consistent painful response (Fig. 4A). Planned comparisons showed that stimulation profiles #4 and #3 enhanced the response (lower NS) compared with both profile #2 (LSD [adjusted 95% CI]  =  −0.62 [−1.03, −0.21], and LSD [adjusted 95% CI]  =  −0.82 [−1.25, −0.39] respectively; adj-*P*<0.001 for both) and #1 (LSD [adjusted 95% CI]  =  −1.00 [−1.40, −0.61], and LSD [adjusted 95% CI]  =  −1.20 [−1.62, −0.79] respectively; adj-*P*<0.001 for both). In addition, profile #2 led to a lower NS than profile #1 (LSD [adjusted 95% CI]  =  −0.38 [−0.73, −0.03]; adj-*P* = 0.031).

The response in OA cats was enhanced (lower NS) compared to the non-OA cats (cat group effect *P* = 0.019): LSD [95% CI]  =  −0.52 [−0.94, −0.10]. In addition, interaction of the stimulation set with the cat group was not significant (*P* = 0.18). This indicated a similar effect across stimulation profiles for each group (equality of the slopes). However, when it (profile of stimulation × group of cat) was tested for the presence of slopes not equal to zero (same model but without the profile effect term), the interaction was significant (*P*<0.001) permitting the following interpretation of planned OA *versus*. non-OA cat comparisons for the different stimulation profiles (Fig. 4B; each stimulation profile was considered independent, implying that no adjustment for multiple comparisons was needed): the response to stimulation profiles #1 and #3 was similar in both OA and non OA cats (LSD [95% CI]  =  −0.20 [−0.69, 0.28], and LSD [95% CI]  =  −0.53 [−1.14, 0.08] respectively; *P* = 0.39, and 0.089 respectively), but NS was lower in OA cats than non-OA cats with stimulation profiles #2 and #4 (LSD [95% CI]  =  −0.76 [−1.26, −0.25], and LSD [95% CI]  =  −0.60 [−1.17, −0.02] respectively; *P* = 0.005, and 0.043 respectively).

There was no significant association between chronic pain measurements and age in any of the above models (PVF, MA intensity, vFWT or stimulation NS) (*P*>0.15). There was a highly significant positive correlation between the vFWT and NS profiles #2 and #4 (Rho_s_ = 0.64, and 0.63, respectively; *P* = 0.01, and 0.02, respectively), but not between PVF or MA intensity and any of the profile responses (all *P*>0.10).

## Discussion

The objective methods PVF, MA intensity and vFWT used in this study to evaluate OA-associated chronic pain enabled non-OA, non painful cats, and painful OA cats to be distinguished. These three evaluation methods were used in a previous study using a larger sample, where both PVF and vFWT discriminated between OA and non-OA cats [10]. Although MA intensity was not sensitive to the presence of OA [10], it was still included in the present study as an objective measure of the effect of OA pain on physical activity and function; MA was significantly affected by both administration of the analgesic non-steroidal anti-inflammatory drug (NSAID) meloxicam [10], [13] and also by feeding an analgesic therapeutic diet [12]. The sample of OA cats used in our study therefore truly characterized cats with OA-related chronic pain. Although the study was slightly underpowered with regards to PVF evaluation, testing the discriminatory ability of PVF in OA cats was not a primary objective.

In the previous study, a four-week NSAID treatment did not eliminate the difference in vFWT between the OA and non-OA cats (OA cats were lower) [10]. Approximately 25% of the OA cats (n = 39) were classified as allodynic based on a repeated duplicate vFWT measurement recorded below a cut-off of 40 g for the front paws and 50 g for the hind paws (first quartile values of the sample of OA cats under placebo) [10]. Most of the OA cats responded favourably to meloxicam, but in those classified as allodynic the response was poor or negligible [10]. This is not surprising in view of the recognized low efficacy of NSAIDs against centralized neuropathic pain [14], [15] and supports the supposition that central sensitization occurs in feline OA-associated chronic pain, similar to humans [15], [20], [21], [23]. While the vFWT was also reliable in OA cats [10], this is primarily only a reflexive evaluation of hypersensitivity [38], [39]. In contrast, evaluation of TS provides the opportunity to evaluate central sensitization with conscious perception since it is based on pain behaviour, implying cortical integration.

We were able to evoke TS in conscious cats, which has not been previously reported in this species. Repetition of sub-threshold mechanical stimuli summated and facilitated pain as detected through observation of pain behaviour, and also detected different responses between OA and non-OA cats. Temporal summation involves conduction of impulses *via* Aδ and C-fibers in wide dynamic range neurons of the dorsal horn, and primarily results from progressive and prolonged dorsal horn C-fibre neuron discharge (windup) [25], [30]. Wind-up and central sensitization are not identical phenomena, but depend on similar pathways, where wind-up initiates and maintains central sensitization [14], [25]. Evoked TS of pain was enhanced (faster) in OA compared to non-OA cats, thereby suggesting that central sensitization plays a role in feline OA-associated chronic pain.

Increasing the stimulus intensity enhanced the cats' response. This is consistent with the supposed mechanism of induced TS, and is in accordance with previous studies [29], [31]. With repeated brief stimuli, the transient first pain response tends to decrease, while second pain increases in intensity and duration, corresponding to prolonged C-fibre discharge [30]. The intensity-dependent response observed in this group of cats suggests that higher intensity stimulation enhanced C-fiber recruitment. The observation of sustained pain behaviours after the end of the stimulation set supports the likelihood that C-fibers were activated. These behaviours persisted for several seconds, consistent with the 15 s aftersensations induced by TS in normal humans [40] and a return to baseline after 30 s in rats [29]. In human patients afflicted with fibromyalgia, aftersensations lasted for up to 120 s after TS of pain was established [40]. This led to the choice of the 5-min delay we imposed between two stimulation sets, preventing persistence of pain into the start of a new stimulation set. Randomization also protected against a potential carry over effect.

Augmentation of stimulation frequency between stimulation profiles #3 and #4 did not affect the time to appearance of pain behaviour, as might be expected [29], [31]. A possible explanation is that the 6N intensity was already close to a single-stimulus pain threshold, so the cats very rapidly experienced pain.

Temporal summation was enhanced in OA cats, particularly with stimulation profiles #2 and #4. This suggests that OA cats with chronic pain have developed central sensitization and the associated pain facilitation. It is noteworthy that the response to both these stimulation profiles correlated positively with the vFWT, supporting the suggestion that profiles #2 and #4 are the best for characterizing central sensitization. The lack of correlation between NS after any stimulation profiles and the other objective evaluation methods of OA-associated disability (PVF and MA intensity) suggests that they can be regarded as complementary assessment methods. This was expected, because TS is specific to central sensitization, which is not correlated with the severity of structural or functional impairment related to chronic pain. The effect of NSAID treatment on MA intensity [10], [13] leads to a similar conclusion, suggesting that MA intensity may be more closely related to the inflammatory component of feline OA pain.

We acknowledge that the cat groups were small and the reported enhancement of mechanical TS in OA cats requires confirmation in a larger study. However, this is the first report of mechanical TS in conscious cats, which was challenging from both a technical and subject acclimation standpoint. Moreover, the use of naturally occurring OA improves the translational potential of these results. This study highlights similarities between cat and human OA-associated chronic pain, which may share similar nociceptive mechanisms. Temporal summation appears to be *N*-methyl-D-aspartate (*N*MDA) receptor-dependent in both animals [41] and humans [42]. Temporal summation QST is a well-recognized mechanism-based evaluation technique for musculoskeletal pain in humans [14], [20], [21], [23]. Hence, evoked TS has considerable potential for effective translational research [43]. A further advantage of investigation into central sensitization is that this phenomenon is potentially reversible. The inefficiency of numerous treatments of human OA-induced chronic pain highlights the need for development of drugs targeting central sensitization (*e.g.*, ionic channel or *N*MDA-receptor blockers, serotonin/noradrenaline reuptake inhibitors) [14]–[17]. The positive results obtained recently in humans using duloxetine, a dual-reuptake inhibitor of serotonin and noradrenaline encourage this approach [44].

In conclusion, our results suggest that central sensitization is a feature of feline OA-associated chronic pain. Use of evoked TS in cats with naturally occurring OA provides a unique opportunity to improve translational research in OA-associated chronic pain, and supports the concept of using naturally occurring disease in animals as an ethical and highly relevant alternative to the use of induced models of pain [45].
