# Correction: Evoked Temporal Summation in Cats to Highlight Central Sensitization Related to Osteoarthritis-Associated Chronic Pain: A Preliminary Study

**DOI:** 10.1371/journal.pone.0114659

**Published:** 2014-11-25

**Authors:** The PLOS ONE Staff

Dr. M. Maxim Moreau is not included in the author byline. He should be listed as the fifth author and affiliated with Groupe de Recherche en Pharmacologie Animale du Québec (GREPAQ), Department of Biomedical Sciences, Faculty of Veterinary Medicine – Université de Montréal, Saint-Hyacinthe, Quebec, Canada and with Osteoarthritis Research Unit, Université de Montréal Hospital Centre, Notre-Dame Hospital, Montreal, Quebec, Canada. The contributions of this author are as follows: performed the experiments, analyzed the data and wrote the paper.

## References

[1] Guillot M, Taylor PM, Rialland P, Klinck MP, Martel-Pelletier J, et al (2014) Evoked Temporal Summation in Cats to Highlight Central Sensitization Related to Osteoarthritis-Associated Chronic Pain: A Preliminary Study. PLoS ONE 9(5): e97347 doi:10.1371/journal.pone.0097347 24859251 10.1371/journal.pone.0097347 PMC4032234

